# SGLT1 contributes to glucose-mediated exacerbation of ischemia–reperfusion injury in ex vivo rat heart

**DOI:** 10.1007/s00395-024-01071-z

**Published:** 2024-08-01

**Authors:** Alhanoof Almalki, Sapna Arjun, Idris Harding, Hussain Jasem, Maria Kolatsi-Joannou, Daniyal J. Jafree, Gideon Pomeranz, David A. Long, Derek M. Yellon, Robert M. Bell

**Affiliations:** 1 Hatter Cardiovascular Institute, Institute for Cardiovascular Science, University College London, 67 Chenies Mews, London, WC1E 6HX UK; 2 Developmental Biology and Cancer Programme, UCL Great Ormond Street Institute of Child Health, London, UK; 3 UCL Centre for Kidney and Bladder Health, London, UK; 4 UCL MB/PhD Programme, Faculty of Medical Sciences, University College London, London, UK

**Keywords:** SGLT1, SGLT2, Myocardial infarction, Reperfusion injury, Diabetes, Hyperglycemia

## Abstract

Hyperglycaemia is common during acute coronary syndromes (ACS) irrespective of diabetic status and portends excess infarct size and mortality, but the mechanisms underlying this effect are poorly understood. We hypothesized that sodium/glucose linked transporter-1 (SGLT1) might contribute to the effect of high-glucose during ACS and examined this using an *ex-vivo* rodent heart model of ischaemia–reperfusion injury. Langendorff-perfused rat hearts were subjected to 35 min ischemia and 2 h reperfusion, with variable glucose and reciprocal mannitol given during reperfusion in the presence of pharmacological inhibitors of SGLT1. Myocardial SGLT1 expression was determined in rat by rtPCR, RNAscope and immunohistochemistry, as well as in human by single-cell transcriptomic analysis. High glucose in non-diabetic rat heart exacerbated reperfusion injury, significantly increasing infarct size from 45 ± 3 to 65 ± 4% at 11–22 mmol/L glucose, respectively (*p* < 0.01), an association absent in diabetic heart (32 ± 1–37 ± 5%, *p* = NS). Rat heart expressed SGLT1 RNA and protein in vascular endothelium and cardiomyocytes, with similar expression found in human myocardium by single-nucleus RNA-sequencing. Rat SGLT1 expression was significantly reduced in diabetic versus non-diabetic heart (0.608 ± 0.08 compared with 1.116 ± 0.13 probe/nuclei, *p* < 0.01). Pharmacological inhibitors phlorizin, canagliflozin or mizagliflozoin in non-diabetic heart revealed that blockade of SGLT1 but not SGLT2, abrogated glucose-mediated excess reperfusion injury. Elevated glucose is injurious to the rat heart during reperfusion, exacerbating myocardial infarction in non-diabetic heart, whereas the diabetic heart is resistant to raised glucose, a finding which may be explained by lower myocardial SGLT1 expression. SGLT1 is expressed in vascular endothelium and cardiomyocytes and inhibiting SGLT1 abrogates excess glucose-mediated infarction. These data highlight SGLT1 as a potential clinical translational target to improve morbidity/mortality outcomes in hyperglycemic ACS patients.

**Supplementary Information:**

The online version contains supplementary material available at 10.1007/s00395-024-01071-z.

## Background

Despite improvements in the management of acute myocardial infarction (AMI), ischemic heart disease remains the leading cause of death worldwide [4]. Among patients with an acute coronary syndrome (ACS), 25–50% will have hyperglycemia at the time of presentation, irrespective of the presence or absence of diabetes mellitus (DM) [10], and recognized as an adverse marker for cardiovascular morbidity [35] and mortality [10]. Epidemiological studies reveal a linear relationship between increasing blood glucose and excess death—a relationship more pronounced in patients without a prior diagnosis of DM [10]. However, demonstration of a causal relationship between glucose and cardiovascular mortality in ACS has proven challenging. The Diabetes Mellitus Insulin Glucose Infusion in Acute Myocardial Infarction (DIGAMI) study [28], a trial of intense glucose control in all ACS patients with a presenting blood glucose greater than 11 mmol/L (198 mg/dL), irrespective of diabetic status, resulted in significant improvements in cardiovascular outcomes. These data suggested that managing hyperglycemia would be beneficial, however, subsequent studies [8, 29] failed to show a similar reduction of cardiovascular mortality. Thus, it remains unclear whether elevated glucose during AMI is directly injurious or simply a biomarker of poor cardiovascular outcome.

While the mechanistic connection between glucose and ACS outcomes remains controversial, the adverse association between glucose and outcomes is recognized in national and international guidelines, with recommendations to control hyperglycemia [9, 32]. However, these guidelines also recognize that aggressive glycemic control is potentially hazardous: hypoglycemia also drives increased mortality [9, 32]. The relationship between blood glucose and mortality is complex, with a dose-response relationship following a “J-curve”: the lowest mortality coinciding with euglycemia in non-diabetic patients (around 5.5 mmol/L), with upward inflections of the mortality curve with both hypo- and hyperglycemia [10].

Sodium/glucose linked transport (SGLT) has emerged as a target for improving outcomes in patients with heart failure where inhibition of the SGLT2 member of the SLC5A solute carrier family reduces hospitalisations and improves cardiovascular outcomes [53]. However, SGLT2 is expressed predominantly in the kidney proximal tubule with lower expression in other tissues and absent in the heart [11, 37]. Thus, the cardiovascular benefits from SGLT2 inhibition are either indirect or via an off-target effector within the myocardium, for which several potential targets have been implicated [2, 7]. The other significant glucose transporter in the SLC5A family is SGLT1. It is expressed within the kidney, but also plays a role in glucose absorption from the gut and is detected in the human heart [42, 51, 52], although its physiological and pathophysiological roles in the myocardium remain to be fully elucidated, but likely include associations with pathological processes such as cardiac oxidative stress, inflammation, fibrosis, and cell apoptosis, as well as mitochondrial dysfunction (see review [51]).

Myocardial SGLT1 expression is upregulated in mouse models of left ventricular dysfunction, hypertrophy and following AMI [39]. Diabetes also alters SGLT1 expression, but reports are conflicting and seemingly dependent upon the model used: Banerjee et al. [3] found that mouse models of streptozotocin-induced type 1 diabetes decreased SGLT1, whereas leptin-deficient *ob/ob* type 2 diabetic mice had increased SGLT1 expression. SGLT1 activity is glucose dependent, and unlike SGLT2 that transports sodium and glucose at a ratio of 1:1, the SGLT1 sodium:glucose ratio is 2:1. Thus, glucose-driven SGLT1 activity leads to greater intracellular sodium influx than SGLT2 under similar conditions. With acute ischemic stress, increased intracellular sodium loading of the cell driven by elevated extra-cellular glucose may imperil viability and survival. This, together with the variances of SGLT1 expression with disease, makes SGLT1 a potential target for understanding the excess injury associated with elevated glucose at the time of AMI.

In this study, we wanted to address five questions: (1) is there a pathophysiological link between glucose at the time of reperfusion and infarct size; (2) in which cells within the myocardium is SGLT1 expressed; (3) is SGLT1 activity, in whole or in part, responsible for excess myocardial injury; (4) does DM alter impact of high glucose upon ischemia–reperfusion injury and (5) does DM induce changes in myocardial SGLT1 expression. To address these study aims, we used a reductionist Langendorff model of reperfusion-adjusted glucose and drug administration following injurious ischaemia and have concentrated on the rat for reasons of availability of the Zucker Fatty Diabetic and Zucker Lean models. We have taken this *ex-vivo* approach for several reasons. First is to isolate the impact of elevated glucose from other metabolic substrates to simplify interpretation, to which a Langendorf model lends itself readily. Second, in focusing the exposure to elevated glucose and/or SGLT inhibitors to the time of reperfusion removes the potential confounder of glucose toxicity either prior to or during ischaemia which may complicate interpretation of the data. Third, there is clinical analogy between this model design and the clinical presentation of an acute coronary artery occlusion that typifies the presentation of ST elevation myocardial infarction (STEMI). In this context, cessation of coronary flow to the distal myocardium by the culprit lesion results in the myocardium in the ischaemic zone being isolated from both systemic stress-induced hyperglycaemia and any parenterally drug administered to manage hyperglycaemia until such time as coronary flow is restored. Thus, a presentation with hyperglycaemic STEMI is a model of reperfusion-glucose injury. If a cardioprotective regimen against hyperglycaemia were dependent upon it being available prior to the onset of ischaemia, its clinical utility in the emergency STEMI setting would be limited.

## Methods

### Animals

Animal use complied with Animals (Scientific Procedures) Act 1986 Amendment Regulations 2012 and was approved by UCL AWERB and UK Home Office. Sprague–Dawley (SD) rats (aged 8–10 weeks, 300–390 g; UCL Biological Services), Goto-Kakizaki (GK) rats (aged 8–10 weeks, 300–390 g; UCL Biological Services), Zucker Diabetic Fatty rats (ZDF) and Zucker lean nondiabetic rats (ZL) (8–10 weeks, 350–450 g; Charles River Laboratories) were used. ZDF rats were fed Purina #5008 diet to induce Type 2 diabetes for four weeks. SD, GK and ZL rats were maintained on Purina #5001 (details of chow given to animals found in supplement section). C57Bl6 male mice (Charles Rover Laboratories), aged 8–10 weeks, body weight 20–25 g were used for the mouse heart dose response study, maintained on standard chow. All animals used were of male gender.

### Reagents

The following SGLT inhibitors were sourced: the specific, but non-selective SGLT inhibitor, phlorizin (Sigma–Aldrich); the SGLT2-selective inhibitor, canagliflozin (Janssen R&D) and the SGLT1-selective inhibitor, mizagliflozin (MedChem Express LLC). Cardiomyocyte isolation/culture reagents: protease XIV and laminin (Sigma–Aldrich), collagenase type 5 (Lorne Labs, UK). Medium 199 and routine biochemicals (Thermofisher Scientific). RNA isolation and PCR kit (Qiagen) and RNAscope^®^ Multiplex Fluorescent Detection Kit (ACD, Biotechne).

### Ex-vivo cardiac ischemia–reperfusion

**Fig. 1:**
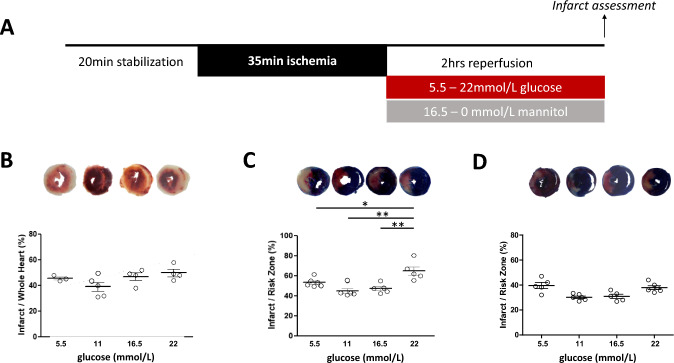
Glucose-infarct size in non-diabetic and diabetic mouse and rat heart models. **A** Protocol for *ex-vivo* experiments. During reperfusion, mannitol is used to ensure that osmolality across different glucose concentration groups remains the same, thus with higher glucose concentration, the mannitol concentration is reciprocally lower. **B** C57BL/6 J non-diabetic mouse hearts (*n* = 4–5/group), subjected to global ischaemia, revealed a dose-range effect with minimum and maximum at 11 mmol/L and 22 mmol/L glucose, respectively. A linear relationship between glucose and infarct size was observed with incremental glucose, with an *r*^2^ = 0.35, *p* = 0.047. **C** SD non-diabetic rat hearts (*n* = 6/group), subjected to regional ischaemia, had minimum and maximum infarction at 11–22 mmol/L glucose, respectively, with a linear relationship between these values (*r*^2^ = 0.56, *p* = 0.0009). **D** GK diabetic rat heart (*n* = 6/group), subjected to regional ischaemia, had a negligible relationship between glucose and final infarct size when compared to non-diabetic hearts, with minimum infarction occurring at 16.5 mmol/L and attenuated peak infarct sizes

*Ex-vivo* perfusion of rodent (mouse and rat) hearts was carried out as previously described [36]. Briefly, animals (either mouse or rat) were anesthetized using pentobarbital sodium (100–150 mg/kg) and anticoagulated with heparin sodium (400–500 u/kg) intraperitoneally. Hearts were harvested and retrogradely perfused with modified Krebs–Henseleit buffer containing 118 mmol/L NaCl, 25 mmol/L NaHCO_3_, 11 mmol/L D-glucose, 4.7 mmol/L KCl, 1.22 mmol/L MgSO_4_·7H_2_O, 1.21 mmol/L KH_2_PO_4_ and 1.84 mmol/L CaCl_2_.2H_2_O (pH 7.4, 37 °C, perfusion pressure 70–80 mmHg). Following 20-min of stabilisation perfusion, hearts were subjected to ischemia for 35 min followed by 2 h reperfusion*.* In the preliminary rat-heart glucose reperfusion experiments (shown in Fig. 1C, D), ischaemia was induced by transient proximal left anterior descending artery ligation and analysis performed as previously described [5]. For mouse heart and all subsequent rat-heart experiments, ischaemia was induced by cessation of coronary flow to induce global ischaemia. The reason for the switch from regional to global ischaemia in the rat model was that we found that the regional ischaemia model did not add any additional information over the global ischaemic model with equivalent ischaemic injury following injurious ischaemia–reperfusion. To save methodological complication and to avoid exclusion of experiments owing to technical issues and to, therefore, minimize animal usage, we concentrated upon the simpler global ischaemic model.

Hearts were randomized to receive reperfusion buffer containing variable glucose concentrations from 5.5 to 22 mmol/L glucose with reciprocal concentration of mannitol (16.5–0 mmol/L) to maintain comparable osmolality between groups (Fig. 1A). In control experiments, vehicle (DMSO, v/v 0.01%) was administered at the time and duration of reperfusion. Where an SGLT inhibitor was used (3 µmol/L phlorizin, 5 nmol/L or 1 µmol/L canagliflozin or 100 nmol/L mizagliflozin), the drug was administered from the onset and duration of reperfusion.

The final concentrations of SGLT inhibitors, phlorin, canagliflozin and mizagliflozin, were selected to act specifically upon SGLT isoforms and avoid off-target affects upon other glucose transportors. Phlorizin’s EC_50_ for SGLT2 and SGLT1 in rat is 75–302 nmol/L, respectively [16], and GLUT transport is only 10% inhibited at 20 µmol/L [16]. Thus 3 µmol/L was used, anticipating inhibition of both SGLT2 and SGLT1 without inhibiting GLUT transport. Canagliflozin is marketed as an SGLT2 inhibitor, but compared to other SGLT2 inhibitors, the relative potency for SGLT2 over SGLT1 inhibition is less, with IC_50_ of 4.2 nmol/L and 663 nmol/L, respectively, in man, and 3.7 nmol/L and 555 nmol/L in rat. The *in-vitro* IC_50_ for canagliflozin against GLUT isoform activity is > 1000 nmol/L [31]. Thus, 5 nmol/L will achieve selective SGLT2 inhibition with 1 µmol/L achieving combined SGLT2 + SGLT1 inhibition, with neither concentration inhibits GLUT glucose transport. Mizagliflozin has an IC_50_ of 166 nmol/L in rat [20] and a Ki of 27.0 nmol/L and 8170 nmol/L for SGLT1 and SGLT2, respectively, from *in-vitro* human studies [19]. Based on these data, we used 100 nmol/to specifically inhibit SGLT1.

At the end of the experiment and after the completion of the reperfusion period, heart infarct size was determined using the tissue viability stain, 2,3,5-triphenyltetrazolium chloride (TTC). The region at risk was identified by re-occluding the LAD in the regional ischaemia studies and injection of Evans blue dye. The area not then stained blue was defined as the area at risk. In global ischaemia models, the whole myocardium was deemed to be the area at risk. Analysis of infarct to area at risk was then determined by blinded digital planimetry analysis using Image J (National Institutes of Health, NIH).

### Isolation of adult rat cardiomyocytes

Cardiomyocyte isolation was based on a previously published protocol with minor modifications [40]. Rat hearts were sequentially perfused with cell isolation buffer (130 mmol/L NaCl, 5.4 mmol/L KCI, 1.4 mmol/L MgCl_2_, 0.4 mmol/L Na_2_HPO_4_, 4.2 mmol/L HEPES, 10 mmol/L glucose, 20 mmol/L taurine, and 10 mmol/L creatine, pH 7.4) containing (1) 750 μmol/L CaCl_2_, (2) 50 μg/ml EGTA, and (3) 100 μmol/L CaCl2 + 50 mg/dL collagenase type 5. Ventricles were minced and triturated with collagenase type 5 (50 mg/dL) and protease XIV (5 mg/dL) with the resulting cells pelleted by gravity. The cell pellet was resuspended in cell isolation buffer containing 1% BSA and increasing concentrations of CaCl_2_ up to a final concentration of 1 mmol/L.

### Tissue collection and processing

Hearts were excised from ZDF and ZL rats under terminal anaesthesia and washed in ice-cold phosphate-buffered saline (PBS). Additionally, kidney, proximal gut and skeletal muscle tissue was harvested to provide control tissue for PCR and RNAscope SGLT expression studies. Kidney was a positive control for both SGLT1 and SGLT2. Proximal gut was a positive control for SGLT1, whereas skeletal muscle was a negative control for SGLT1. For RT-PCR, tissue samples were stored in RNAlater™. For analysis of tissues by immunohistochemistry and RNAscope, samples were fixed in 4% paraformaldehyde overnight, washed with PBS and stored in 70% ethanol until processed for paraffin-embedding and sectioning.

### Polymerase chain reaction (PCR)

RNA was extracted from rat (SD, ZDF, ZL) whole heart and kidney tissue and rat primary isolated cardiomyocyte cells using RNeasy Mini Kit and concentration and purity measured by OD at 260 nm and 269/280 nm ratio using LVIS plate (BMG Labtech). Following cardiomyocyte primary isolation, RNA extraction was also undertaken on the non-cardiomyocyte fraction.

RNA (1 µg) was reverse-transcribed into cDNA using oligo dT Primers and an RT–PCR Kit. PCR was performed for (1) *Sglt1* exon 1–3, forward primer 5’-ACCGCCATGGACAGAGCACCT-3’ [position 122–143], reverse primer 5’- GCGTTCCATTCGAAGCCACCCA-3’ [position 420–441], with a product of 320 bp; (2) *Sglt1* exon 3/4–5, forward primer 5’-GCTTCGAATGGAACGCCTTG-3’ [position 426–445], reverse primer, 5’-TCCAAATCGCTTCCGCAGAT-3’ [position 519–538], product size 113 bp; (3) *Sglt1* exon 8–9, forward primer 5’-GAATGTTACACACCCAGGGC-3’ [position 887–906], reverse primer, 5’-AGACATGTTCTTGGCCGAGA-3’ [position 1032–1051], product size 165 bp; (4) *Sglt1* exon 13–15 (forward primer 5’-TGCGGGGTCCACTATCT-3’[position 1733–1749], reverse primer 5’-CAACTCCAGAGTCGCCA-3’ [position 2191–2207], product size 475 bp) and (5) *Sglt2* (forward primer 5’-TTCTGTCATCGCACTCTTGG-3’ [position 527–546], reverse primer 5’-GATCCTTGGACACCGTCAGT-3’ [position 716–735], product size 209 bp). As housekeeping genes, either *Gapdh* (forward primer 5’- TGATGGGTGTGAACCACGAG-3’ [position 464–483], reverse primer 5’- AGTGATGGCATGGACTGTGG-3’ [position 596-615 bp], product size 152 bp), or *Hprt*, (forward primer 5’-ACGTTCTAGTCCTGTGGCCATC-3’ [position 785–806], reverse primer: 5’-ATCAAAAGGGACGCAGCAACAG-3’ [position 869–890], estimated product size 106 bp) were used.

### RNAscope

RNAscope on formalin-fixed paraffin-embedded (FFPE) tissue Sects. (5 μm) from ZDF and ZL rat were performed using the Multiplex Fluorescent Assay kit as per manufacturer’s instructions. Sections were hybridized with probe-sets targeting *Sglt1* or *Sglt2* mRNA and counterstained with 4′,6-diamidino-2-phenylindole (DAPI). Positive and negative probes were used as controls. Co-detection of *Sglt1* and *Sglt2* mRNA with anti-CD31 antibodies (Abcam; ab222783,1:150) followed by Goat Anti-Rabbit IgG H&L (Alexa Fluor^®^488; ab150081,1:300) or the cell marker Wheat Germ Agglutinin (WGA), Alexa Fluor^™^ 488 Conjugate (Invitrogen^™^; W11261,1:150) was performed according to manufacturer’s instructions. For semiquantitative analysis of RNA signals, the number of DAPI-stained nuclei and *Sglt* mRNA signals were ascertained in five random microscopic fields of each section using Image J. Average RNA signals for each tissue section were determined by dividing the total section *Sglt* mRNA signal count by total section nuclei count.

### Immunohistochemistry

Immunohistochemical probing of FFPE sections was carried out as previously described [26] with a primary antibody against SGLT1 (ab14685, Abcam) and counterstained in Mayers hematoxylin.

### Single-nucleus RNA-sequencing analysis of human transcripts

To profile the expression of SGLT-encoding in human heart transcripts, to ascertain whether the SGLT profile observed in rat heart is similar to that in man, we analyzed two publicly available single-nucleus RNA-sequencing (snRNA-seq) datasets sourced from the Gene Expression Omnibus (GSE183852) [23] and the Broad Institute’s Single Cell Portal (https://singlecell.broadinstitute.org/single_cell/study/SCP498/transcriptional-and-cellular-diversity-of-the-human-heart) [43]. Analyses were performed in R (v4.0.2), using the Seurat package [17]. An object was created from each dataset, containing donors without evidence of overt cardiac disease, with associated metadata including biological sex or age group of the donors [23] and cardiac chamber from which the cells originated from [43]. Cardiomyocyte count matrices were normalized and scaled before principal component analysis, with donor integration using Harmony [24], shared nearest neighbour graphing, and unsupervised clustering. Transcript enrichment was visualised using Feature plots or Violin plots and quantified using differential expression tests. The code used to perform analysis can be accessed at: https://github.com/daniyal-jafree1995/collaborations/blob/main/Almalki%26Arjun_SGLT.R.

### Statistical analysis

Differential expression tests for snRNA-seq data utilized Wilcoxon Rank Sum tests in RStudio. All other analyses were performed using GraphPad Prism version 6. Normality was assessed using the Shapiro–Wilk test. Normally distributed data are presented as mean ± SEM and analyzed by unpaired *t*-test for two independent groups and ANOVA with Tukey’s multiple comparison test for three or more independent groups. Preliminary experiments were performed to ascertain the relationship between myocardial injury and glucose concentration (Fig. 1). Based on these data, which informed effect size and standard deviation, power calculations were performed a priori, to calculate group size in subsequent studies, based on achieving a power of 80% and an *α* of 0.05. *p* value of < 0.05 was considered significant.

## Results

### Elevated glucose during reperfusion leads to increased myocardial infarct size

We performed ischaemia–reperfusion experiments using Langendorff-perfused hearts to examine how altering the glucose content of the reperfusion buffer affected infarct size. Initially, we undertook a preliminary dose-ranging study utilising glucose concentrations based on published epidemiological data of blood glucose levels in patients presenting with an ACS, ranging from < 3.9 mmol/L (< 70 mg/dL) to > 22 mmol/L (> 370 mg/dL) [10], with cross-group osmolality maintained with mannitol (Fig. 1A).

In both C57Bl6 mice and SD rats (Fig. 1B, C, respectively), the smallest infarct size was observed when the non-diabetic heart was reperfused with 11 mmol/L glucose (mouse: 38.7 ± 3.4% versus 49.2 ± 3.2% at 22mmol/L; rat data below), consistent with previous published observations in Langendorff-perfused rat heart [33]. At glucose concentrations below 11 mmol/L, infarct size tended to increase. At higher concentrations, infarct size increased linearly with increasing glucose, with maximum injury observed at 22 mmol/L, with significant Pearson correlation coefficients and coefficients of determination with *r*^2^ of 0.35 and 0.56 (*p* < 0.05 and *p* < 0.001) in mouse and rat, respectively (Fig. 1B, C). The observed effect size and spread of data enabled us to design and power subsequent experiments to investigate the role of elevated glucose in this increased infarct size based on the minima and maxima observed.

While the preliminary mouse data (Fig. 1B) were under-powered for ANOVA analysis of significance between infarct sizes observed at individual glucose concentrations, the rat data were powered to enable inter-group comparison. In SD rat, 22 mmol glucose significantly increased infarct size (64.8 ± 4.2%), compared to 16.5 mmol/L (47.4 ± 2.1%; *p* < 0.01), 11 mmol/L (44.7 ± 2.6%; *p* < 0.01) and 5 mmol/L (53.2 ± 1.8%; *p* < 0.05).

Next, we examined whether these findings were replicated in the setting of diabetes, using the GK rat, that had a mean fasting glucose of 12.11 ± 3.03mmol/L (Table S1). Interestingly, the relationship between glucose concentration and infarct size was absent in diabetic GK rat hearts (Fig. 1D), indicating relative resistance to elevated glucose compared to non-diabetic heart. While the smallest infarcts in non-diabetic SD rat were observed at 11 mmol/L glucose (44.7 ± 2.6%), in diabetic GK rat, this occurred at 16.5 mmol/L (25.6 ± 3.4%). Infarcts in the GK rat tended to be smaller than SD rat at each glucose concentration, reaching statistical significance when comparing 16.5 mmol/L (25.6 ± 3.4% versus 47.4 ± 2.1%; *p* < 0.01) and 22 mmol/L (36.7 ± 5.4% versus 64.8 ± 4.2%).

### Expression of SGLT transcripts in rat myocardium: SGLT1 but no SGLT2

**Fig. 2:**
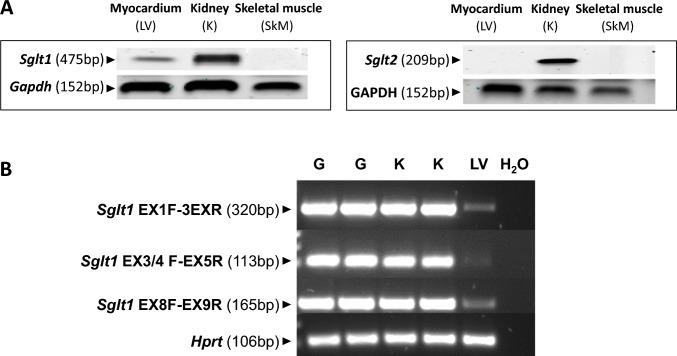
SGLT1 and SGLT2 expression in healthy rat and human myocardium. **A** *Sglt1* and *Sglt2* rtPCR in rat myocardium (left ventricle, LV), kidney (K, positive control) and skeletal muscle (SkM, negative control). In heart *Sglt1* (using primers to detect exon 13–15), but not *Sglt2* mRNA is detected (*n* = 5–9, representative blots shown). *Gapdh*: housekeeping gene, Glyceraldehyde-3-phosphate dehydrogenase. **B** Agarose gel electrophoresis depicting PCR amplification products for the rat *Sglt1* gene using primers to detect exons 1–3, 3–5 and 8–9. DNA fragments of the expected size for *Sglt1* are visible. Lane labels: G-gut, K-kidney, LV-left ventricle, each correspond to individual rat samples. *Hprt*: housekeeping gene, Hypoxanthine Guanine Phosphoribosyl Transferase

We hypothesized that SGLT1 might contribute to the effect of high glucose on infarct size. To explore this further, we firstly assessed rat tissue expression of both *Sglt1* and *Sglt2* mRNA, using kidney as positive and skeletal muscle as negative control tissues. Using primers to detect exon 13–15, we found SD rat hearts expressed transcripts encoding for *Sglt1* (Fig. 2A and Fig. S1). Furthermore, myocardial *Sglt1* expression was qualitatively less than that seen in kidney, consistent with published data [6]. As prior studies have shown that the mouse and human heart only express *Sglt1* transcripts consisting of exon 9–15 which is predicted to result in a non-functional, truncated cardiac *Sglt1* protein [15], we designed further primers to detect exon 1–3, 3–5 and 8–9 of rat *Sglt1*. Contrary to the previously published mouse data, positive signals were also detected using these primers, indicating the presence of the earlier SGLT1 exons in the rat heart (Fig. 2B). We did not detect any transcripts for *Sglt2* within the rat heart (Fig. 2A and Fig.S1).

### SGLT1 distribution within human myocardium

**Fig. 3:**
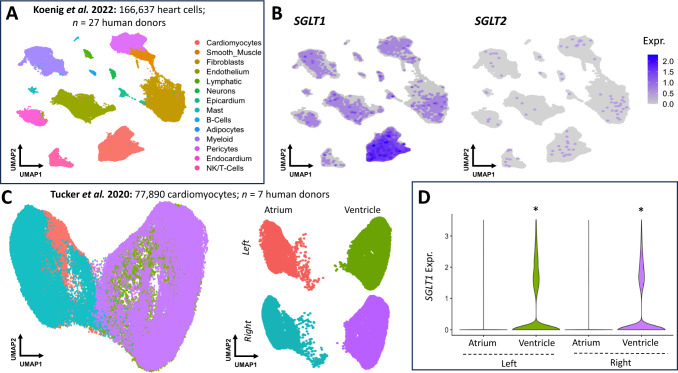
Human cellular expression of SGLT1.** A** Uniform manifold approximation and projection (UMAP) of snRNA-seq data of human heart cells from Koenig et al. 14 distinct cell types were identified, including cardiomyocytes. **B** *SGLT1* expression (left UMAP) is enriched in cardiomyocytes. Conversely, *SGLT2* expression (right UMAP) is scant. **C** UMAP of snRNA-seq data human cardiomyocytes (top UMAP) from Tucker et al. The bottom UMAP represents stratification of cardiomyocytes into the four chambers, with atrial and ventricular cardiomyocytes resolving separately. Analysis of the relative expression of *SGLT1* in atrial *versus* ventricular cardiomyocytes (**D**)

While whole heart SGLT1 expression has been reported [3], SGLT1 distribution within the myocardium by cell type is poorly understood. To address this question, we initially determined the cellular expression profiles of SGLT-encoding transcripts within the human heart, leveraging two published snRNA-seq datasets. One dataset comprised of 166,637 heart cells featuring fourteen cell types from 27 donors [23]. Figure 3A reveals cardiomyocyte enrichment for *SGLT1* (average log fold change; log_2_FC = 0.64, adjusted *p* < 0.0001) with concomitant albeit lower expression in non-cardiomyocytes (predominantly of vascular origin: endothelium, pericytes and smooth muscle, Fig. 3B). *SGLT2* expression was scant in the human heart (Fig. 3B). To validate this in an independent dataset, we interrogated snRNA-seq data of 77,890 atrial or ventricular cardiomyocytes from seven human donors [43] with *SGLT1* but not *SGLT2* expressed, the former enriched within cardiomyocytes (log_2_FC = 1.02, adjusted *p* < 0.0001). Donors within the datasets were stratified by biological sex, age or heart chamber of origin. Our analyses demonstrated some variability of myocardial *SGLT1* expression across individuals at different age groups, albeit with subtle but statistically significant enrichment in male donors (log_2_FC = 0.13, adjusted *p* = 1.3 × 10^–16^, Fig. 3C). Moreover, *SGLT1* was enriched in ventricular compared to atrial cardiomyocytes in the right and left heart (Fig. 3D).

### RNAscope and immunohistochemistry

**Fig. 4:**
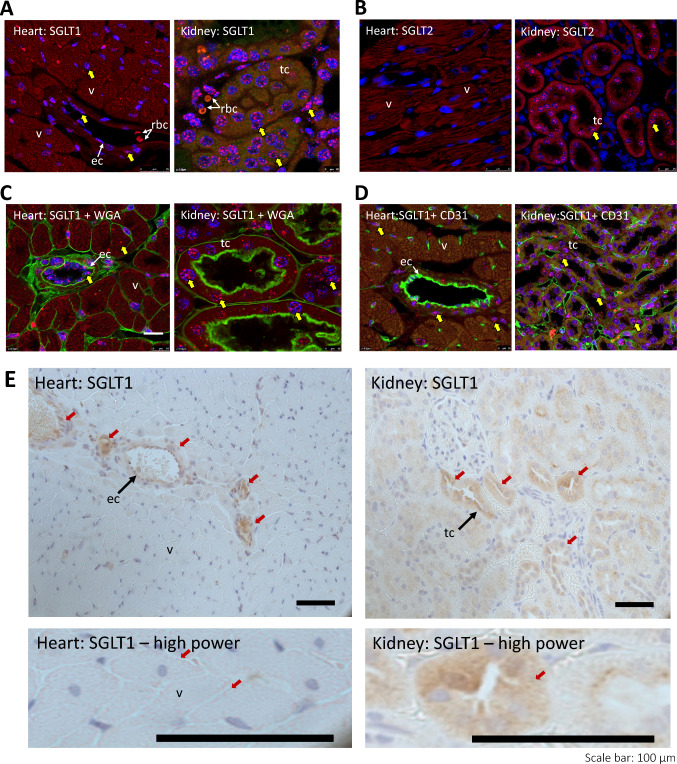
SGLT1 and SGLT2 expression in the SD rat myocardium. **A** RNAscope revealed *Sglt1* mRNA expression in both cardiomyocytes (which auto-fluoresce red), and vascular structures. Kidney is a positive control, with a strong SGLT1 signal (red) within the proximal nephron. *v* ventricular cardiomyocyte, *ec* endothelial cell, *rbc* red blood cell, *tc* tubular cell. Yellow arrows highlight SGLT detection signal: here predominantly in DAPI stained nuclei showing red/magenta signal. **B** There was no SGLT2 signal detectable within the heart, in contrast to kidney. Vascular markers, cell membrane marker, WGA (green) (**C**), and endothelial marker CD31 (green) (**D**) were used to show SGLT1 distribution. **E** Immunohistochemistry for SGLT1 protein expression (brown) reveals similar cellular staining pattern in heart and kidney to that seen with RNAscope. In the heart, staining can be found in the sarcolemma of the cardiomyocytes and the endothelium of arterioles and capillary structures (highlighted with red arrows)

RNAscope was then used to visualize SGLT1 expression in the rodent heart and kidney (probe negative and positive controls together with small intestine and skeletal muscle as positive and negative tissue controls shown in Fig.S3, 4, 5). We found that the whole myocardium had clearly demonstrable *Sglt1* expression. In accord with our snRNA-seq data *Sglt1* was predominately detected within cardiomyocytes, but also in the vasculature (Fig. 4A). No myocardial expression was observed with *Sglt2*, although there is clear expression in the kidney (Fig. 4B). The vascular expression of *Sglt1* appears to be predominantly endothelial, as determined with counterstaining with WGA and CD31 cellular markers (Fig. 4C, D). Immunohistochemistry for SGLT1 protein, with an SGLT1 antibody raised against a synthetic peptide corresponding to human SGLT1 amino acids 600–700, resulted in staining patterns consistent with the mRNA/ RNAscope data, with positive signals observed within vascular structures and cardiomyocytes (Fig. 4E).

### Impact of non-selective SGLT inhibition upon glucose associated excess injury

**Fig. 5:**
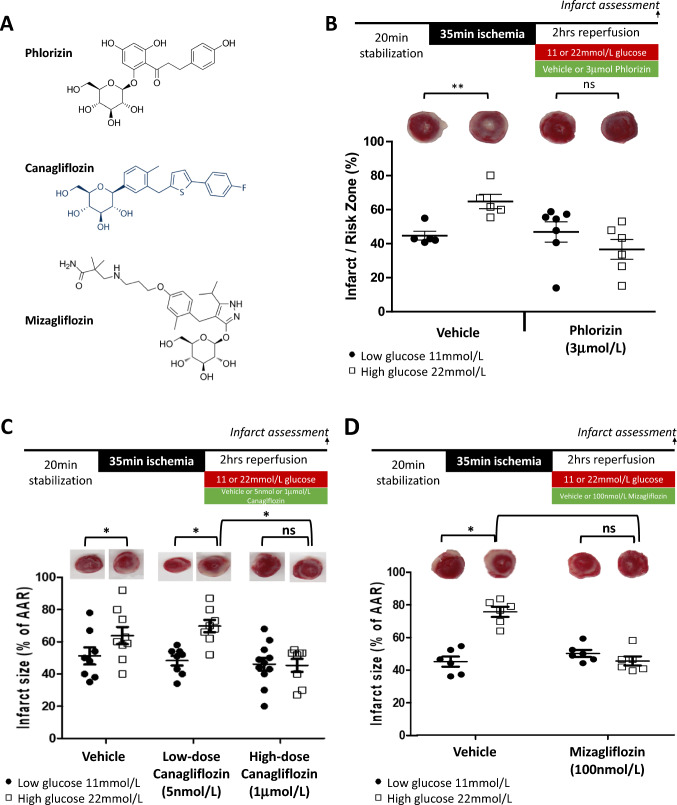
Inhibition of SGLT2 and SGLT1 in 11 and 22 mmol/L glucose at reperfusion. **A** Chemical structures of the SGLT inhibitors used. **B** In non-diabetic SD rat heart, elevated glucose at reperfusion results in a significant increase in infarct over size the control condition which is completely abrogated by the administration of phlorizin (3 µmol/L). **p < 0.001 22 mmol/L versus 11 mmol/L reperfusion glucose. **C** There is a significant increase in infarct size with high (22 mmol/L) glucose versus 11 mmol/L, which is not ameliorated by 5 nmol/L canagliflozin. However, 1 µmol/L canagliflozin abrogates this excess injury. **D** Mizagliflozin, abrogates the excess injury associated with elevated glucose. Representative mid-ventricular myocardial slices shown. *p < 0.05 versus respective control

Given that there is widespread vascular and cardiomyocyte expression of SGLT1 in the heart, we hypothesized that inhibition of SGLT1 would abrogate excess myocardial injury associated with elevated glucose upon reperfusion. We utilized three chemically and pharmacologically distinct inhibitors: a specific, non-selective SGLT inhibitor phlorizin, the relatively specific SGLT2 inhibitor, canagliflozin and the SGLT1-specific inhibitor, mizagliflozin (Fig. 5A).

To assess the impact of SGLT inhibition upon infarct size, phlorizin (3 µmol/L) was added with either elevated (22 mmol/L) or standard glucose (11 mmol/L + 11 mmol/L mannitol) at the time of reperfusion. As previously mentioned, the EC_50_ of phlorizin in rat for SGLT2 and SGLT1 is 75 and 302 nmol/L, respectively [16], and GLUT transport is only 10% inhibited at 20 µmol/L [16], thus 3 µmol/L would be expected to inhibit both SGLT2 and SGLT1 without significant impact upon GLUT transport. To determine the size of our experimental groups, an a priori power calculation was performed based on our preliminary SD rat dose-ranging experiments demonstrating a 40% absolute increase in infarct size from 11 to 22 mmol/L glucose, with the hypothesis that phlorizin would abrogate this increase (representing an effect size of 20 with a standard deviation of 10, with four groups).

22 mmol/L high glucose, led to a significant 44% increase in infarct size versus the 11 mmol/L control heart, an increased that was completely abrogated by the administration of phlorizin (65 ± 4.2% to 37 ± 5.8% in 22 mmol/L with and without, phlorizin respectively, *p* < 0.01, Fig. 5B). Phlorizin had no impact upon infarct size under “standard” glucose conditions of 11 mmol/L (45 ± 2.6% *versus* 47 ± 5.8% in control versus phlorizin).

### Selective SGLT1 versus SGLT2 inhibition

As previously described, canagliflozin has an IC50 of 3.7 nmol/L and 555 nmol/L for SGLT2 and SGLT1 in rat respectively and an *in-vitro* IC_50_ against GLUT of greater than 1000 nmol/L [31]. Thus, 5 nmol/L will achieve selective SGLT2 inhibition with 1 µmol/L achieving combined SGLT2 + SGLT1 inhibition, and neither concentration inhibits GLUT glucose transport. We hypothesised that SGLT2-specific dose canagliflozin would not impact infarct size, whereas 1 µmol/L, inhibiting both SGLT1 and SGLT2, would abrogate the excess injury associated with high glucose during reperfusion. As part of our statistical design, we took the data from our phlorizin study to refine and make a new calculation of group size. To achieve an 80% power and an *α* of 0.05, we calculated a sample size of *n* = 9 per group to determine a difference of 25%.

In vehicle controls, high glucose increased infarct size (from 51 ± 5.3% to 64 ± 5.3%, *p* < 0.05, Fig. 5C). 5 nmol/L canagliflozin failed to ameliorate the increase in infarction associated with high glucose (48 ± 2.9% versus 70 ± 3.8% in 11 and 22 mmol/L glucose respectively, *p* < 0.001), however,,,,,, 1 µmol/L canagliflozin completely abrogated the excess injury associated with 22 mmol/L glucose (45 ± 4.0% versus 46 ± 4.0% for 11 and 22 mmol/L glucose respectively, *p* = 0.99, Fig. 5C, representative heart slices Fig.S6). Thus, specific SGLT2 inhibition had no impact upon the excess injury associated with high glucose, whereas combined SGLT2 + SGLT1 inhibition abrogated this deleterious effect.

In the absence of demonstrable SGLT2 expression within the heart, we postulated that the high-dose 1 µmol/L canagliflozin mediates its protective effect against high-glucose mediated injury via myocardial SGLT1 inhibition. However, to explore this further, we tested a specific SGLT1 inhibitor, mizagliflozin. As previously described, mizagliflozin has an IC_50_ of 27 nmol/L for SGLT1 [12] (versus 8170 nmol/L for SGLT2). Thus, 100 nmol/L mizagliflozin will inhibit SGLT1 specifically, and in doing so, we hypothesized that it would completely abrogate glucose-mediated excess injury. We subsequently found that 22 mmol/L glucose increased infarct size relative to 11 mmol/L (65 ± 2.6% compared to 39 ± 2.4%, *p* < 0.001), but this excess injury was abrogated by mizagliflozin (43 ± 1.9% versus 39 ± 2.4% in 11 and 22 mmol/L glucose respectively, *p* = 0.65, Fig. 5D, representative heart slices Fig. S7).

### Impact of diabetes upon SGLT1 and SGLT2 expression in the myocardium

Injury resulting from high glucose at the time of reperfusion is absent in diabetic heart (Fig. 1D). SGLT2 kidney expression increases with diabetes but published data reports are conflicting as to whether diabetes up- or down-regulates myocardial SGLT1 [3]. Additionally, the impact of diabetes upon myocardial SGLT2 expression is unknown.

**Fig. 6:**
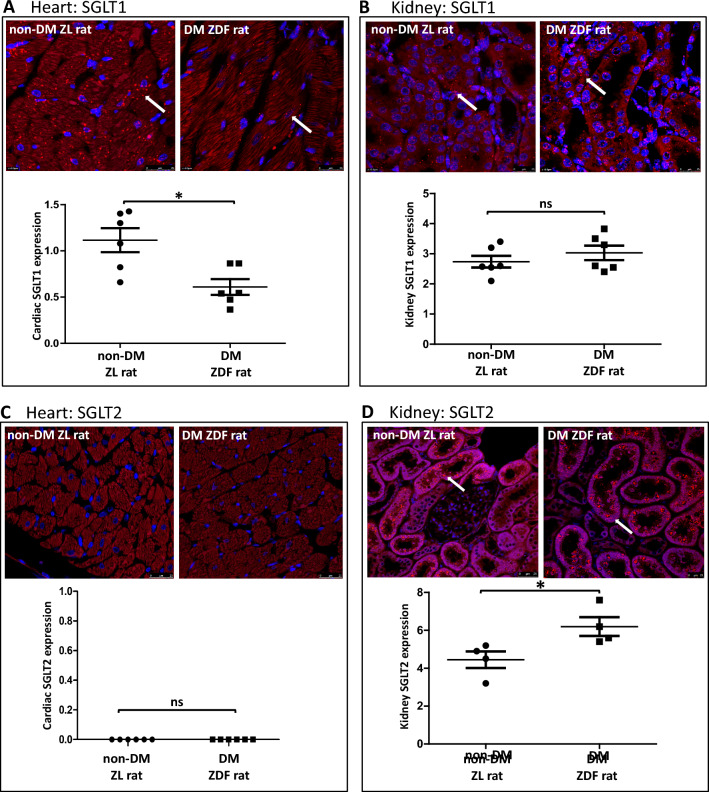
SGLT1 and SGLT2 expression in non-diabetic and diabetic heart. **A** In ZL rat heart, myocardial *Sglt1* mRNA expression was markedly higher than that seen in ZDF rat heart (*n* = 6/group). **B** *Sglt1* mRNA expression in kidney was not significantly altered in the ZDF diabetic model. **C** Diabetes did not result in a compensatory increase of *Sglt2* mRNA expression. **D** Renal *Sglt2* expression is significantly increased in diabetic kidney versus non-diabetic kidney. *p < 0.05

Therefore, myocardial SGLT expression was investigated by RNA-scope using ZDF and ZL rat hearts with the same animals’ kidney as a positive control tissue. ZDF rats were diabetic, with a mean random glucose of 31.52 ± 1.06 mmol/L compared to non-diabetic ZL rat, 7.87 ± 0.61 mmol/L (Table S1). In the ZDF rat hearts, *Sglt1* expression was 50% less than in non-diabetic ZL heart (*p* < 0.05, Fig. 6A). In contrast, renal *Sglt1* expression in ZDF and ZL rats were similar (Fig. 6B). Conversely, *Sglt2* expression remained absent in diabetic heart (Fig. 6C) but was significantly increased by 50% in diabetic kidney relative to non-diabetic animals, as previously reported [46] (Fig. 6D).

## Discussion

In this study, we demonstrate that reperfusion with elevated glucose results in a glucose dose-dependent increase in myocardial infarct size in non-diabetic mouse and rat hearts, whereas the diabetic rat heart was resistant to the adverse impact of high glucose upon reperfusion. As has previously been demonstrated, we find that rat heart expresses SGLT1 and, using snRNA-seq datasets, detected *Sglt1* expression within human ventricular myocardium, particularly endothelium and cardiomyocytes. Using RNAscope, we demonstrate the distribution of *Sglt1* expression throughout the rat myocardium, with prominent expression in endothelium as well as cardiomyocytes, in line with the human transcriptome analysis. Using three chemically distinct SGLT inhibitors, with differing SGLT1/ SGLT2 specificity, we found that SGLT1 but not SGLT2 protects against the adverse impact of raised glucose at reperfusion, and that the relative resistance of diabetic rat to raised glucose at reperfusion correlated to low SGLT1 myocardial expression in the diabetic heart.

The observed pattern of SGLT1 expression likely represents a novel coronary glucose extraction and distribution system within the heart [38]. In this experimental model of myocardial infarction, however, this transport mechanism becomes pathological, with excess glucose-driven myocardial injury in non-diabetic heart that is completely abrogated by pharmacological SGLT1 inhibition. Interestingly, we observe that the heart of diabetic rat is relatively resistant to high glucose: the excess myocardial necrosis with raised glucose is absent, which appears to correlate with significantly lower SGLT1 expression in the diabetic heart. These data appear to parallel epidemiological patient mortality data, with a similar relationship between glucose concentration and outcome [10] namely, as with infarct size in diabetic rat, that mortality with glucose is substantially lower in those with diabetes. The similarity may be coincidental, but given that larger infarcts are associated with higher mortality following AMI [41], a clear and testable translational hypothesis can be derived: effectively managing glucose, particularly in patients without prior known diabetes, should result in improved cardiovascular outcomes and reduced mortality through reduced myocardial injury.

### SGLT1 inhibition is protective only against excess injury from elevated glucose

Inhibiting SGLT1 abrogates the excess myocardial injury associated with elevated glucose upon reperfusion. However, under standard isolated perfused heart conditions, with a glucose of 11 mmol/L, SGLT1 inhibition has no impact upon infarct size. This observation is consistent with our prior study using canagliflozin in both the diabetic ZDF and non-diabetic ZL rat [25]. We and others have shown that SGLT2 inhibition can trigger protection in the heart when the drug is administered *in-vivo* irrespective of pre-existing diabetes or circulating glucose concentration via cardioprotective kinase activation [25, 30], whereas *ex-vivo* administration of SGLT2 inhibitors to isolated heart with perfusate containing 11 mmol/L glucose fails to reduce infarct size [22, 25, 45].

Interestingly, we found that the cardioprotection against high-glucose mediated excess myocardial injury was achieved without lowering the circulating glucose level; the glucose concentration was fixed by the formulation of the perfusate. Therefore, an SGLT1 inhibitor can be cardioprotective against hyperglycaemia even prior to any anticipated reduction in circulating glucose levels. This is exciting as a translational observation: one needs only to achieve a plasma concentration of the SGLT1 inhibitor to achieve protection and not need to wait for glucose elimination.

### Translatability

The other advantage of SGLT inhibitors is the lack of hypoglycaemia resulting from their use. Hyperglycemia is common in ACS and is poorly managed [34, 48], at least in part owing to concerns of inducing hypoglycemia, because hypoglcemia is also associated with increased mortality (see review [13]). Current glucose management strategies in AMI tend employ insulin are thus prone to cause hypoglycemia unless scrupulously monitored [34]. Thus, SGLT1 inhibitors offer the potential for safely mitigating the risks associated with elevated glucose. As mentioned above, SGLT1 inhibitors protect the myocardium even in the absence of immediate glucose lowering, and when they do lower glucose, they do not cause hypoglycaemia. Therefore, inhibiting SGLT1 has two beneficial modes of action: a rapid, direct anti-glucotoxic effect and a slower, secondary glucose-lowering effect through glucose renal excretion and inhibited gut absorption without the risk of hypoglycaemia.

### Infarction in the diabetic heart

The diabetic heart is resistant to high glucose (Fig. 1D). Smaller infarcts in diabetic versus non-diabetic heart is well recognized under normal glucose conditions, which we and others have reported and discussed previously, and appears related to chronic activation of cardioprotective signalling pathways, characterized by Akt-phosphorylation [49]. Reduced infarct size is a phenomenon also seen in diabetic patients [14], but these patients are prone to poorer outcomes despite the smaller initial infarct. In addition to a smaller infarct size under the 11 mmol/L starting glucose, we found that, in contrast to the non-diabetic SD rat, increased glucose at reperfusion did not result in a significant increase in infarct size in the diabetic GK rat heart. Thus, the diabetic heart has greater tolerance to high glucose levels.

SGLT1 appears pivotal for glucose-mediated injury in non-diabetic heart, it is, therefore, notable that in our model, the diabetic heart had significantly lower SGLT1 expression. While SGLT1 inhibition did not reduce infarct size at 11 mmol/L glucose in non-diabetic heart, it did abrogate the excess injury associated with high, 22 mmol/L glucose. In the diabetic heart, we found that it had no such excess increase in myocardial injury with high glucose, but the diabetic heart had down-regulated SGLT1 expression. Thus, the diabetic heart has self-reduced SGLT1 expression and potentially also function that is likely directly related to the observed resistance to glucose-mediated myocardial injury.

The mechanism by which SGLT1 expression is suppressed in diabetes is unclear, but it is attractive to postulate that its expression is regulated to some degree by circulating glucose levels; a process by which high circulating glucose down-regulates SGLT1 in response to energy “plentifulness”. In pancreatic islet cells, glucose and cAMP have been shown to dephosphorylate the ser-275 of TORC2 leading to CREB activation [21], and that TORC2:CREB is linked to genomic SGLT1 expression. Whether such a mechanism of SGLT1 expression regulation occurs within the heart would be an interesting area for future investigation.

### SGLT1 expression in disease states

Under the pathophysiological conditions of AMI, SGLT1 downregulation protects the diabetic heart against injurious impact of elevated glucose. Several studies have reported that SGLT1 expression is altered by disease, including diabetes. However, there is controversy in this area: Banerjee et al. demonstrated reduced SGLT1 expression in a streptozotocin-induced type 1 diabetic mouse model, whereas they found increased expression in the *ob/ob* obese mouse model of type 2 diabetes [3]. Our SGLT1 expression data are consistent with their type 1 but not their type 2 diabetic model. The explanation for the latter is unclear: the *ob/ob* type 2 diabetic mouse is similar to ZDF rat, with both models having leptin signalling deficiency. However, the ZDF rats in our model have no evidence of heart failure, and it is not clear whether the *ob/ob* mice in Banerjee’s study had preserved function; they compare their data with those patients with end-stage heart failure with type 2 diabetes, and it is recognised that both heart failure (from a diabetic cardiomyopathy for example) or elevated leptin levels are both associated with increased SGLT1 expression [3]. Had we left our ZDF rats for longer with poorly controlled diabetes, it would be interesting to see whether secondary compensatory up-regulation of SGLT1 would emerge, resulting from the development of a diabetic cardiomyopathy.

### Clinical trial implications of SGLT1 expression in disease states

Differential expression of SGLT1 in diabetics versus non-diabetics has important implications in clinical trial design and interpretation. A large diabetic population will both reduce the expected excess myocardial injury and attenuate the benefit of any glucose-moderating intervention: the trial would need appropriate patient stratification and be powered accordingly. Our data also suggest that glucose-mediated injury occurs rapidly (within two hours of reperfusion), so delays in achieving effective glucose lowering, if not targeting SGLT1 directly, would likely lead to failure to protect the heart. This may in part explain why intensive insulin therapy studies such as DIGAMI-2 and HI-5 failed to achieve expectations, with neither trial achieving anticipated glucose lowering targets (DIGAMI-2—fasting glucose target 5–7 mmol/L, achieved 8.0 mmol/L; HI-5 achieved mean 8.3 ± 2.2 mmol/L with mean duration of symptom onset to commencement of insulin of 13 h, thus well after the onset of reperfusion). Moreover, given that heart failure and elevated leptin levels increase SGLT1 expression means that comorbidities also have potentially significant clinical importance: hearts expressing more SGLT1 would be predicted to be more vulnerable to the adverse impact of elevated glucose at the time of AMI and thus such patients would likely benefit more from timely SGLT1 inhibition. In clinical trial design, these factors would, therefore, need to be accounted for and stratified during patient recruitment.

### Pharmacological inhibition of SGLT: off target effects and mechanisms

The current study was not designed to look directly into the mechanism beyond the involvement of SGLT1. In the field of SGLT2 inhibition, unlike SGLT1 inhibition, there is data for cardioprotection even under normal glucose conditions. Under these circumstances, a variety of mechanisms have been suggested, including inhibition of the sodium hydrogen exchange inhibitor 1 (NHE1) [44], or through high-glucose mediated reactive oxygen species generation through activation of SMIT1 [47]. However, if protection was mediated through NHE1, one would expect protection both under normal glucose conditions, even in isolated hearts, but neither we nor others, in similar isolated perfusion models, observe such protection [22, 25, 45]. In respect to the involvement of SMIT1, this certainly needs to be further investigated, but given that the SMIT IC_50_ for the SGLT inhibitors used (eg canagliflozin [27]) is over five times higher than the highest dose used in this study, and the dose of phlorizin in the SMIT1 investigation [47] is a thousand-fold higher than used in our study (and that other published data suggest that 500 μmol/L of phlorizin – over 100 times used in our study-is required to suppress 90% of SMIT1 activity [1]), it is not clear whether these potential pathways are likely explanations for the protection that we have observed.

A further issue that we need to consider is that raised by Ferte et al. [15], namely that in mouse and human, there is evidence of truncated, non-functional forms of SGLT1. In the mouse, the group was the first to describe the truncated form of SGLT1, missing exons 1–8. It was not clear whether such a truncated form of SGLT1 was also present in our rat model. We therefore undertook rtPCR studies looking at exons 1–3, 3–5 and 5–9, and found expression of each suggesting that there is indeed a functional SGLT1 expression within rat heart, consistent with the RNAscope and immunohistochemistry data that we present. Further work is required to explore the precise levels of non-truncated and truncated SGLT1 in the rat heart and whether this balance is functionally important.

### Study limitations

Our study has a reductionist experimental design using the *ex-vivo* isolated Langendorff heart perfusion model giving us full control over glucose concentration, the timing of raised glucose and duration of ischemia and onset of reperfusion. With crystalloid perfusates, such as the modified Krebs-Henesleit buffer used here, there are no concerns regarding the viscosity and thus flow of the perfusate through the coronary vasculature. However, plasma viscosity is a likely contributing factor in microvascular obstruction and no-reflow would be a concern in the whole animal and in patients [50]. The problem with this reductionist model, however, is that there are no other metabolic substrates available nor endocrinological factors that regulate metabolic substrate utilisation, such as insulin. The next logical experimental step would, therefore, be an *in-vivo* model, although reproducing stress-hyperglycemia is not straightforward. Stress hyperglycemia remains relatively understudied and, therefore, adequately and reproducibly replicating these conditions is difficult. A possible avenue is intravenous administration of glucose, but this may have compensatory impacts upon circulating insulin and gut hormone release, that may have unpredictable effects upon myocardial injury and SGLT1 expression and is unlikely to reproduce the expected insulin resistance. However, given published data that infarct size correlates directly to mortality [41] and that our reductionist model data show that infarct size increases with higher glucose concentrations upon reperfusion with similarity to the glucose/mortality relationship seen in epidemiological data, suggests that the model assumptions made are appropriate and presents a platform for further studies.

## Conclusion

Our novel findings are that elevated glucose, at the time of reperfusion following an injurious ischemic insult, results in a biologically important 45% increase in myocardial injury and infarction, and that this can be pharmacologically inhibited with an SGLT1 inhibitor. Moreover, we demonstrate the vascular endothelial and cardiomyocyte distribution of SGLT1. Interestingly, the glucose-mediated excess of reperfusion injury is marked in non-diabetic heart but is significantly attenuated in the diabetic heart, which appears correlated with suppressed diabetic myocardial SGLT1 expression. Our data, therefore, suggest an exciting opportunity to re-purpose existing SGLT1 inhibitors to further improve outcomes in ACS with concomitant hyperglycemia, and is consistent with calls for adjuvants to reperfusion therapy for patients with multiple morbidities presenting with AMI [18].

## Supplementary Information

Below is the link to the electronic supplementary material.

Supplementary file1 (DOCX 5868 KB)

## Data Availability

The authors confirm that the data supporting the findings of this study are available within the article [and/or] its supplementary materials. Raw data were generated at University College London. Derived data supporting the findings of this study are available from the corresponding author [RMB] on request. Human snRNA-seq data sets are available on-line from references provided.
