# Supplementary material for: SGLT1 contributes to glucose-mediated exacerbation of ischemia–reperfusion injury in ex vivo rat heart
Source: Basic Res Cardiol. 2024 Aug 1;119(5):733–49. doi: 10.1007/s00395-024-01071-z (PMC11461679; doi:10.1007/s00395-024-01071-z)
Supplement: Supplementary file 1 — Supplementary file1 (DOCX 5868 KB) [file 395_2024_1071_MOESM1_ESM.docx]

**Supplement figure 1: Representative image of RT-PCR expression in myocardium, kidney and skeletal muscle.** SGLT1 mRNA expression was detected in the heart and kidney (positive control), but not in skeletal muscle (negative control) (n=6-10 animals).


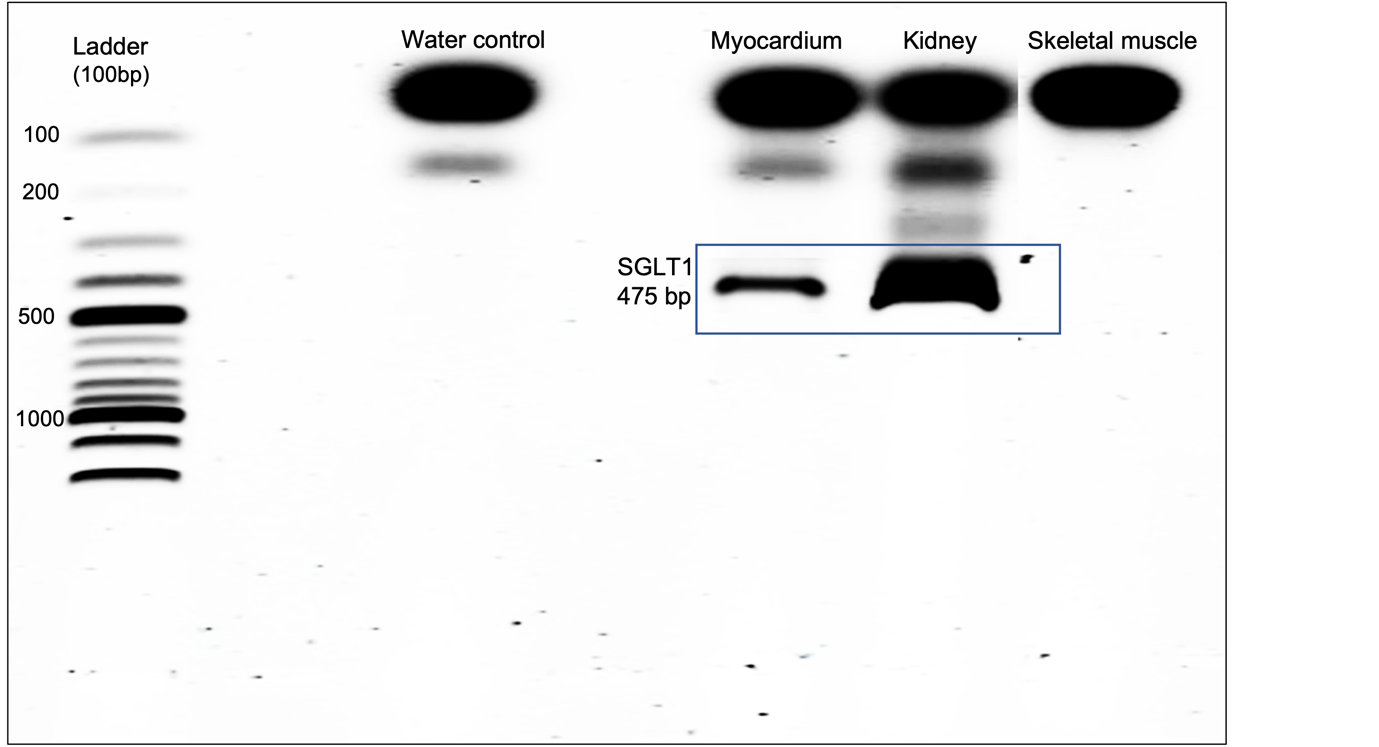


**Supplement figure 2: Representative RT-PCR of SGLT1 expression in cardiac chambers.** SGLT1 mRNA is detected in all heart chambers, kidney used as a positive control (n=10 animals).


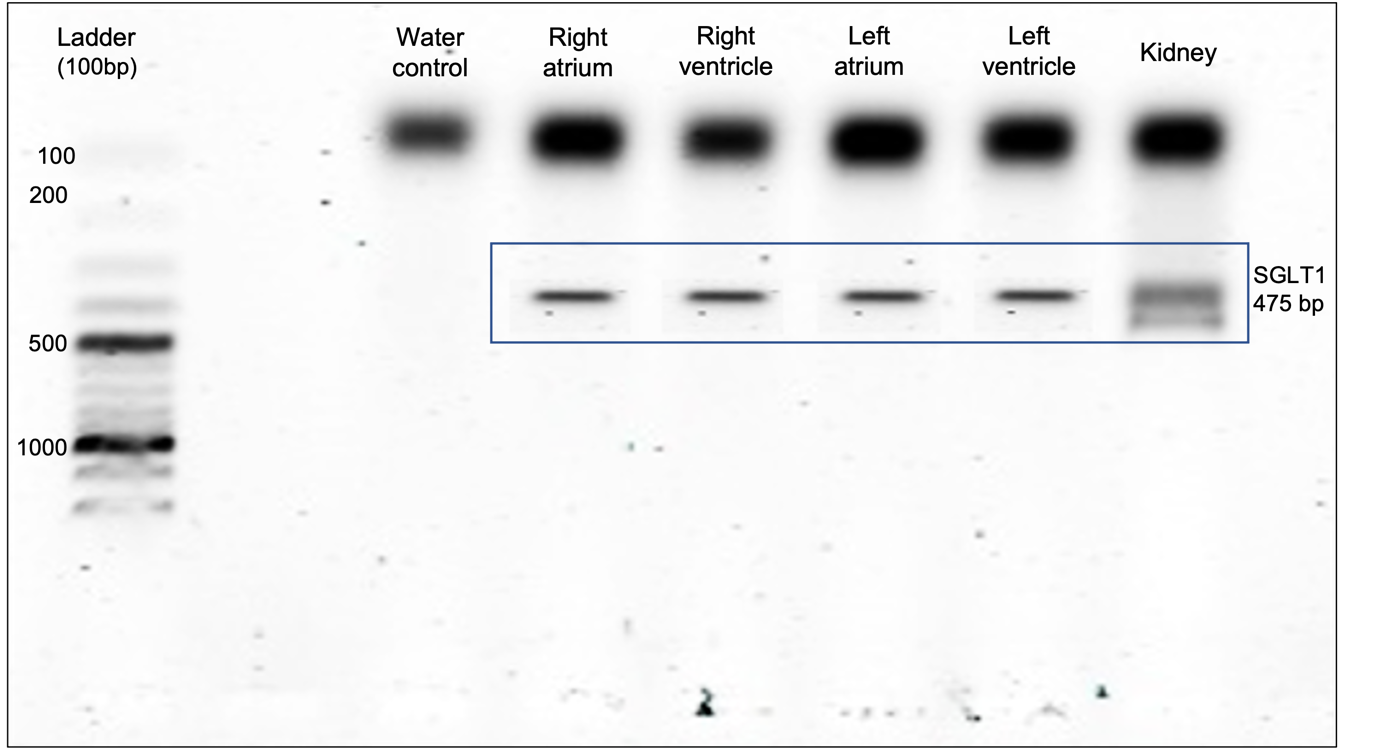


**Supplement figure 3: Representative positive and negative RNAscope mRNA control probe images. Panel A:** negative control probe targeting bacterial DapB. **Panel B:** positive control probe targeting PPIB mRNA (red signal). The nucleus is stained with DAPI (blue). With the negative probe (panel A), there is no staining in the heart tissue (only red signal is from background autofluorescence), but the positive control (panel B) produces significant staining of PPIB in the heart.


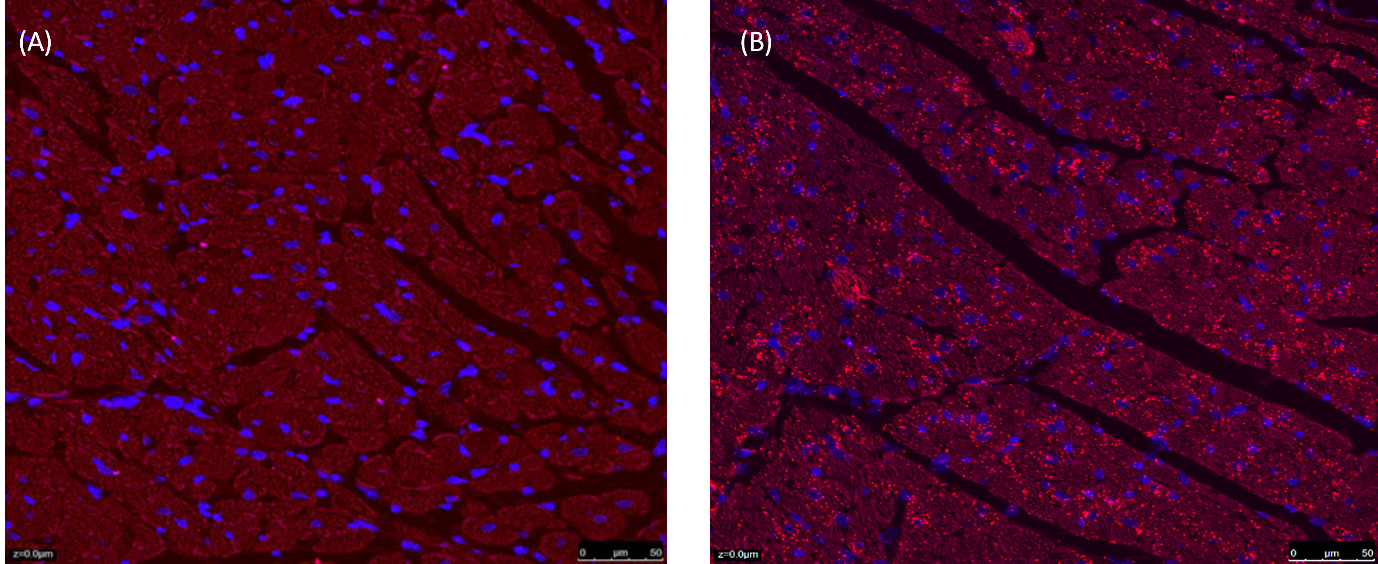


**Supplement figure 4: SGLT1 positive control tissue – proximal ileum**. Representative images of RNAscope of SGLT1 mRNA expression in proximal ilium of Sprague Dawley rats (panels A and B). The arrow indicates the SGLT1 mRNA signal (red fluorescent dots -highlighted with white arrows) on the autofluroscent red background (n=6 animals). Consistent with published literature, there is robust SGLT1 mRNA expression in the ileal villi epithelium.


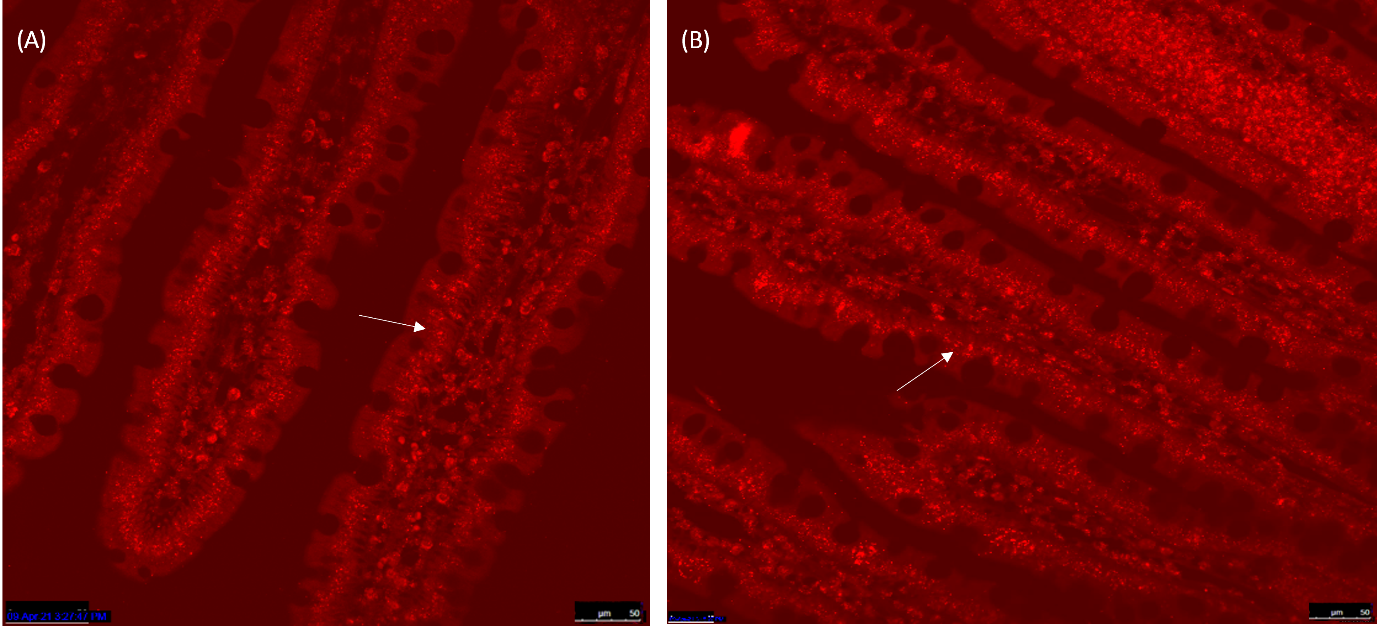


**Supplement figure 5: SGLT1 negative control tissue – skeletal muscle.** Representative RNAscope image of SGLT1 mRNA expression in the skeletal muscle of Sprague Dawley rats (panels A and B). Consistent with published literature, there was no SGLT1 mRNA in skeletal muscle (n=5 animals).


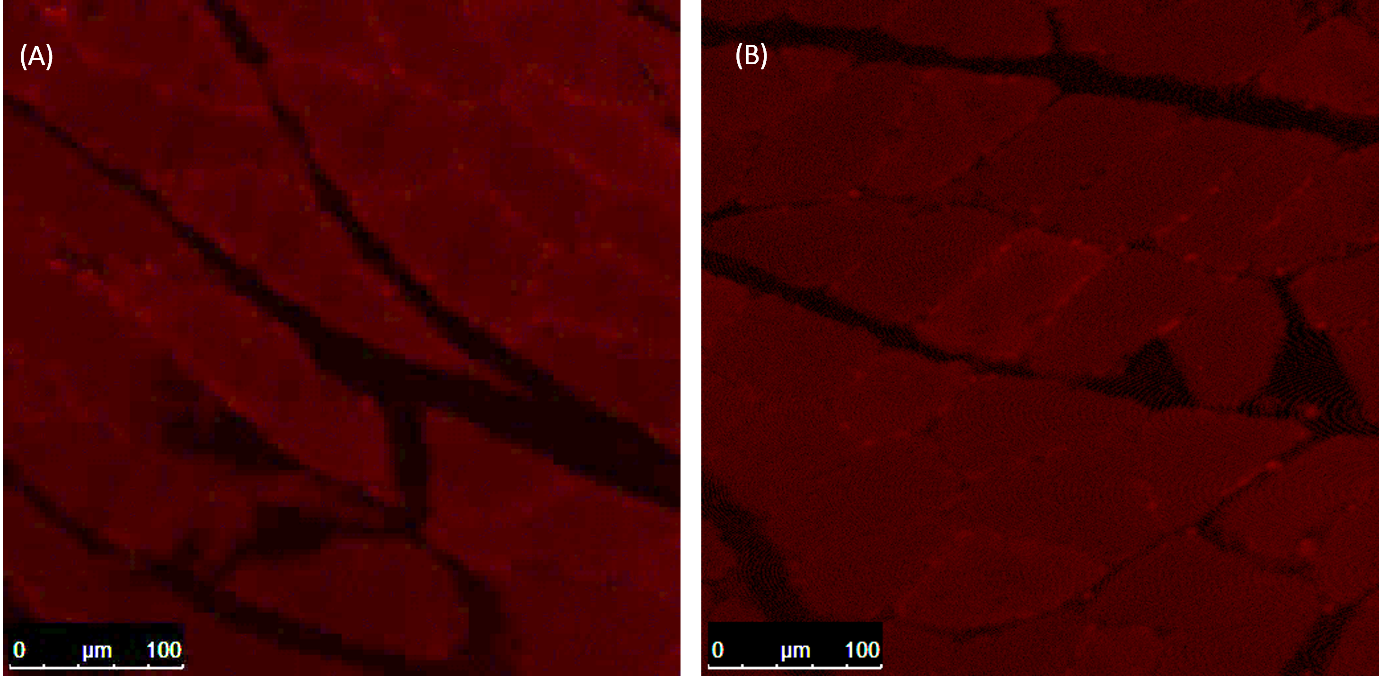


**Supplement figure 6: Representative Sprague Dawley heart slices from canagliflozin treatment**

**experiments in both standard 11mmol/L glucose + 11mmol/L mannitol (panels A-C) and high 22mmol/L glucose (panels D-F).** **Panels A & D:** DMSO treated heart. **Panels B & E:** Low dose (5nM) Canagliflozin heart. **Panels C & F:** High dose (1uM) canagliflozin heart.

**Standard glucose (11mmol/L + 11mmol/L mannitol) [11mM]**

**Standard glucose [11mM]**


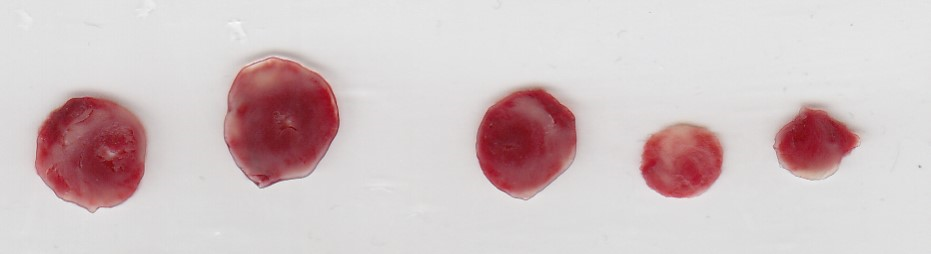

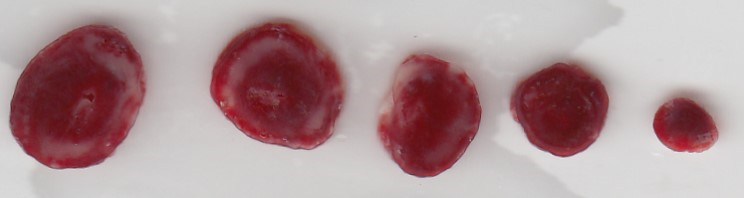

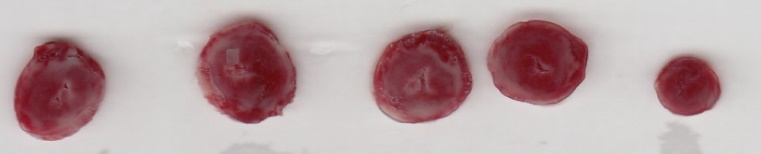


1. DMSO treated control heart
2. DMSO treated heart

(B) 5nM canagliflozin treated heart

1. 5nM canagliflozin treated heart

(C) 1uM canagliflozin treated heart

1. 1uM canagliflozin treated heart


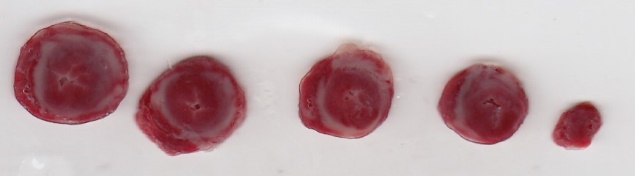

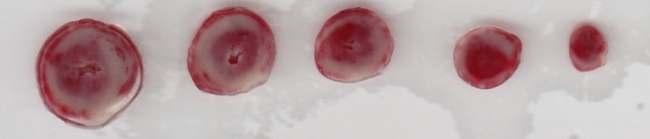


(D) DMSO treated control heart

(D) DMSO treated heart

(E) 5nM Canagliflozin treated heart

(E) 5nM Canagliflozin treated heart

(F) 1uM canagliflozin treated heart

(F) 1uM canagliflozin treated heart


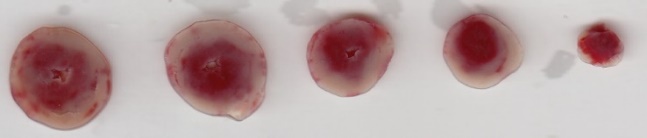


**High glucose (22mmol/L)**

**High glucose [22mM]**

**Supplement figure 7: Representative Sprague Dawley heart slices from mizagliflozin treatment experiments in both standard 11mmol/L glucose + 11mmol/L mannitol (panels A & B) and high 22mmol/L glucose (panels C & D). Panels A & C:** DMSO treated control heart. **Panels B & D:**  Mizagloflozin treated heart. Inhibiting SGLT1 with mizagliflozin Showed abrogation of the infract size with high glucose [22mM].

**High glucose [22mM]**

**High glucose [22mM]**


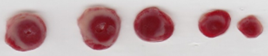

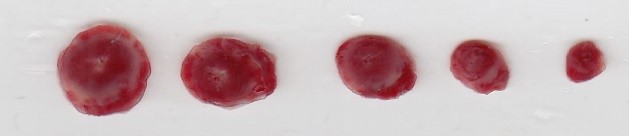


(C) DMSO treated control heart

(C) DMSO treated heart

(D) Mizagliflozin treated heart

(D) Mizagliflozin treated heart


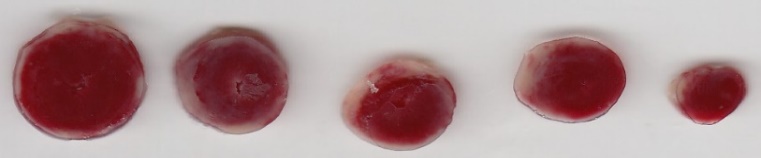

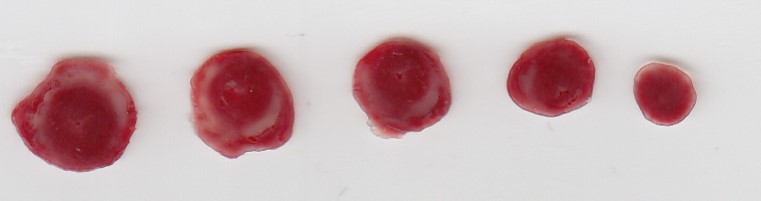


**Standard glucose [11mM]**

**Standard glucose [11mM]**

(B) Mizagliflozin treated heart

(B) Mizagliflozin treated heart

(A) DMSO treated control heart

(A) DMSO treated heart

**Table S1** – age range, weight and fasting glucose characteristics of rats

| Species/strain | Age (weeks) | Body weight (g) | Fasting glucose (mmol/L) |
| --- | --- | --- | --- |
| Rat/ Sprague Dawley | 8-10 | 358±22 | 7.04±0.18 |
| Rat/ Goto-Kakizaki | 8-10 | 416±29 | 12.11±3.03 |
| Rat/ Zucker lean | 8-10 | 294±17 | 7.87±0.61 |
| Rat/ Zucker diabetic fatty | 8-10 | 393±9 | 31.52±1.06 |
